# Changes in survival and characteristics among older stroke unit patients—1994 versus 2012

**DOI:** 10.1002/brb3.1175

**Published:** 2018-11-25

**Authors:** Angela Susan Labberton, Ole Morten Rønning, Bente Thommessen, Mathias Barra

**Affiliations:** ^1^ Health Services Research Unit Akershus University Hospital Lørenskog Norway; ^2^ Institute of Clinical Medicine University of Oslo Lørenskog Norway; ^3^ Department of Neurology Akershus University Hospital Lørenskog Norway

**Keywords:** cerebrovascular disorders, mortality, risk factors, stroke

## Abstract

**Objectives:**

Treatment on organized stroke units (SUs) improves survival after stroke, and stroke mortality has decreased worldwide in recent decades; however, little is known of survival trends among SU patients specifically. This study investigates changes in survival and characteristics of older stroke patients receiving SU treatment.

**Materials & Methods:**

We compared 3‐year all‐cause mortality and baseline characteristics in two cohorts of stroke patients aged ≥60 consecutively admitted to the same comprehensive SU in 1994 (*n* = 271) and 2012 (*n* = 546).

**Results:**

Three‐year survival was 53.9% in 1994 and 56.0% in 2012, and adjusted hazard ratio (HR) was 0.99 (95% CI: 0.77–1.28). Adjusted 30‐day case fatality was slightly higher in 2012, 18.9% versus 16.2%, HR 1.68 (95% CI: 1.14–2.47). There were no significant between‐cohort differences in survival beyond 30 days. Patients in 2012 were older (mean age: 78.8 vs. 76.7 years) and more often admitted from nursing homes. There were higher rates of atrial fibrillation (33.7% vs. 21.4%) and malignancy (19.2% vs. 8.9%), and prescription of antiplatelets (46.9% vs. 26.2%) and warfarin (16.3% vs. 5.5%) at admission. Stroke severity was significantly milder in 2012, proportion with mild stroke 66.1% versus 44.3%.

**Conclusions:**

Three‐year survival in older Norwegian stroke patients treated on an SU remained stable despite improved treatment in the last decades. Differences in background characteristics may explain this lack of difference; patients in 2012 were older, more often living in supported care, and had higher prestroke comorbidity; however, their strokes were milder and risk factors more often treated.

## 1 INTRODUCTION

Stroke mortality has decreased worldwide during the last two decades (Feigin et al., 2014), but it is uncertain which factors have contributed. During recent decades, stroke units (SUs) have become established in many countries; the benefits of which are now well proven (Stroke Unit Trialists' Collaboration, 2013) since the first controlled trials were conducted in the 1990s (Indredavik et al., 1991; Ronning & Guldvog, 1998a, 1998b). SU treatment improves both survival and function, with patients treated at an SU having better outcomes compared to treatment on alternative wards (Stroke Unit Trialists' Collaboration, 2013). Better access to SUs (Addo et al., 2011; Appelros et al., 2014) may have contributed to better outcomes; however, changes in patient risk profile and other characteristics have likely contributed more at the population‐level (Feigin et al., 2014).

Existing studies reporting trends in stroke risk factors or outcomes include either both hospitalized and nonhospitalized patients (Feigin et al., 2015; Feigin, Lawes, Bennett, Barker‐Collo, & Parag, 2009; Lecoffre et al., 2017; Lee, Shafe, & Cowie, 2011; Numminen, Kaste, Aho, Waltimo, & Kotila, 2000; Rothwell et al., 2004; Wieberdink, Ikram, Hofman, Koudstaal, & Breteler, 2012), or patients treated in hospital, but not necessarily on an SU (Appelros et al., 2014, 2010; Arboix et al., 2008; Carrera, Maeder‐Ingvar, Rossetti, Devuyst, & Bogousslavsky, 2007; Nimptsch & Mansky, 2014). Several do not examine changes in survival outcomes (Bembenek et al., 2015; Lecoffre et al., 2017; Teuschl et al., 2013; Wieberdink et al., 2012), and none of these papers report on long‐term survival. We were interested in whether there have been changes in survival among patients receiving SU treatment specifically. SU treatment is the gold standard treatment for stroke patients, and there have been some medical advances in recent years that have been shown to improve outcomes. Management on an SU is the gold standard treatment for stroke patients, and medical advances in recent years have been shown to improve outcomes. Insight into any changes in survival, stroke severity and other patient and disease‐related characteristics in the SU patient population would be relevant for clinicians working in this setting and may also help interpret secular trends in stroke outcomes in the general population.

The aim of this study was to investigate changes in survival following SU treatment by comparing two cohorts of stroke patients treated on the same comprehensive SU two decades apart. Secondary objectives were to investigate changes in stroke severity and other baseline characteristics in this population.

## 2 MATERIALS AND METHODS

### 2.1 Study setting and participants

Akershus University Hospital is situated in the Oslo greater metropolitan area and serves approximately 10% of Norway's population. National policy is to admit all suspected strokes as early as possible and without prior medical examination. Emergency medical services are publically funded in Norway; therefore, patients within the catchment area are admitted to the hospital without any prior selection. Data on consecutive stroke admissions to the SU were prospectively collected from 1 March 1994 to 31 December 1995 (the 1994 cohort), and from 15 February 2012 to 15 March 2013 (the 2012 cohort). The 1994 cohort forms one arm of a quasi‐randomized, controlled study investigating the effect of SU versus general medical ward care (Ronning & Guldvog, 1998a, 1998b) and is described elsewhere (Ronning & Guldvog, 1998b). The 2012 cohort is a subset of the Norwegian Stroke—Paths of Treatment (NOR‐SPOT) cohort (Barra, Simonsen, & Dahl, 2016), collected to investigate delivery of health services to Norwegian stroke patients. NOR‐SPOT includes all admissions to the dedicated SU, plus the few additional stroke patients admitted elsewhere due to overcrowding, but received treatment by the same stroke physicians regardless.

The inclusion criteria used in 1994 were applied to the 2012 cohort for comparability: Patients were included if they received SU treatment, were discharged with a diagnosis of stroke, presented within 24 hr of symptom onset (one calendar day in 2012), and were aged ≥60 years.

The WHO definition of stroke (WHO Task Force on Stroke and other Cerebrovascular Disorders, 1989) was used; however, patients diagnosed with stroke but with symptom resolution within 24 hr due to treatment with intravenous thrombolysis were still classified as having stroke (five cases). Both first‐ever and recurrent strokes were included, but only the first admission during the study period.

### 2.2 The stroke unit

The SU was representative of a typical Scandinavian‐model comprehensive SU (Langhorne & Pollock, 2002) in both 1994 and 2012. It is a dedicated ward with 10 beds in 1994 and 29 beds in 2012. In 2012, four were located in an intensive monitoring area. The catchment area comprised 291,905 persons in 1994 (49,303 aged ≥60 years) and 498,697 in 2012 (96,920 aged ≥60) (Official Statistics of Norway, 2018). The large increase in persons was due to the inclusion of three Oslo boroughs in 2011, which led to an increase of 45% to the catchment area. The current practice guidelines were utilized (Committee for medical technology evaluation at the Norwegian Research Council, 1995; Norwegian Directorate of Health, 2010), and the recommended features of SU care (Stroke Unit Trialists' Collaboration, 2013) were present at both time points. The unit is described in detail elsewhere (Ronning & Guldvog, 1998a, 1998b).

The main difference between 1994 and 2012 was the use of intravenous thrombolysis and endovascular interventions. In 1994, there was no recommendation for thrombolysis outside of clinical trials. In 2012, recanalization treatment (primarily thrombolysis) was considered in patients presenting within 4.5 hr and without contraindications. Thrombectomy, while available in 2012, was performed on only five patients.

Physiological parameters were systematically and intermittently registered; in 2012, continuous monitoring was available on indication. Other differences include improved imaging techniques, including MRI angiography and more use of ultrasound, and the availability of newer oral medications in 2012 versus 1994. Surgery for carotid stenosis was generally performed within 2 weeks of the stroke in 2012 versus 6 months in 1994. Combination antithrombotic therapy or monotherapy with clopidogrel was routinely prescribed in 2012, as were lipid‐lowering agents. Patients in the 1994 cohort were quasi‐randomized to supplemental oxygen or not for the first 24 hr as part of an additional study; (Ronning & Guldvog, 1999) however, the treating physician was instructed to administer oxygen on indication regardless of randomization.

### 2.3 Outcomes and measures

The primary outcome in this study was all‐cause mortality during the 3 years (1,080 days) following index stroke. Mortality information was obtained via the official national register.

Stroke severity on admission was scored prospectively by the neurologist on duty. In the 1994 cohort, the Scandinavian Stroke Scale (SSS) (Scandinavian Stroke Study Group, 1985) was used, and in 2012 the National Institutes of Health Stroke Scale (NIHSS) (Brott et al., 1989). Both scales are reliable and valid (Askim, Bernhardt, Churilov, & Indredavik, 2016; Goldstein & Samsa, 1997; Lindenstrøm & Boysen, 1991). Where a prospective NIHSS score was unavailable, the first author (ASL) scored patients retrospectively using admission records and a validated algorithm (Williams, Yilmaz, & Lopez‐Yunez, 2000). For between‐cohort comparability, the scales were trichotomized using previously published, comparable cut‐offs (Askim et al., 2016; Bernhardt et al., 2015; Govan, Langhorne, & Weir, 2009) into mild (NIHSS: 0–7; SSS: 43–58), moderate (NIHSS: 8–16; SSS: 26–42), and severe (NIHSS: 17–42; SSS: 0–25). Primary stroke type was determined via imaging (ischemic vs. hemorrhagic). Reduced consciousness at admission was defined by a score of ≤2 on item 1 of the SSS in 1994 and by a Glasgow Coma Scale score of ≤10 in 2012, as these were deemed to be equivalent. Length of stay was recorded.

Patient demographics, medication use and the following comorbidities and stroke risk factors were registered at admission: previous cerebrovascular disease (transient ischemic attack or stroke), myocardial infarction, treated hypertension or diabetes, any cancer diagnosis, and current smoking status. Atrial fibrillation was considered present if previously diagnosed or shown on electrocardiogram during admission.

Level of function poststroke was graded using the Barthel Index (BI) (Mahoney & Barthel, 1965) or modified Rankin Scale (mRS) (Swieten, Koudstaal, Visser, Schouten, & Gijn, 1988). BI was scored in 1994, and either BI or mRS in 2012. The scales are highly correlated (Uyttenboogaart, Luijckx, Vroomen, Stewart, & Keyser, 2007), and patients were classified as being independent by either BI score ≥85 or mRS score ≤2. These cutoffs are shown to be comparable (Sulter, Steen, & Keyser, 1999; Uyttenboogaart et al., 2007) and are commonly used to indicate “good outcome.” Function was scored on day 4 or 5 of admission in 1994, and in 2012 on median day 4 (interquartile range [IQR]: 2–6).

### 2.4 Statistical analysis

Continuous variables are presented as mean and standard deviation (*SD*), or median and IQR, and categorical variables as frequency and percentage. Independent samples *t* tests were used to test differences between continuous outcomes, chi‐squared tests for categorical outcomes. Adjusted odds ratios (ORs) were estimated using logistic regression. Survival analysis was performed using Kaplan–Meier survival plots and the log‐rank test for between‐group differences. Hazard ratios (HRs) were estimated using Cox regression for the entire follow‐up time, and in a subanalysis of different time points and intervals poststroke. We allowed a minimum of ten events‐per‐variable for adjusted HRs. Variables entered into the models were selected based on current knowledge, without using a computerized selection process. We were unable to construct fully adjusted models for ischemic and hemorrhagic stroke separately due to the study's moderate sample size; however, we included a dummy ICH variable in the overall Cox regression model to partially compensate for this while still retaining power, and also constructed smaller adjusted Cox regression models for each stroke type separately, the results of which were not significant. Due to relatively few events at 7 days, we limited adjustments in the subanalysis to the seven variables, including cohort, we deemed most relevant. Assessments for collinearity, missed confounding due to baseline differences, and the proportional hazards assumption were performed and found to be satisfactory. SPSS version 21 was used for all analyses.

### 2.5 Ethical considerations

Collection of the 1994 cohort data was approved by the Regional Committee for Medical and Health Research Ethics (REC; approval number S‐93231), and consent obtained prior to recruitment. The NOR‐SPOT project was classified as a quality assurance project by REC so ethical approval was granted by the Data Protection Officer at Akershus University Hospital (approval number 11‐076), in accordance with REC's recommendation. Permission for the use of data from 1994 for the current study was deemed by REC to fall outside of its mandate since the data were fully anonymized. Consequently, approval for its use was granted by the hospital's Data Protection Officer under NOR‐SPOT's existing approval number.

## 3 RESULTS

**Figure 1:**
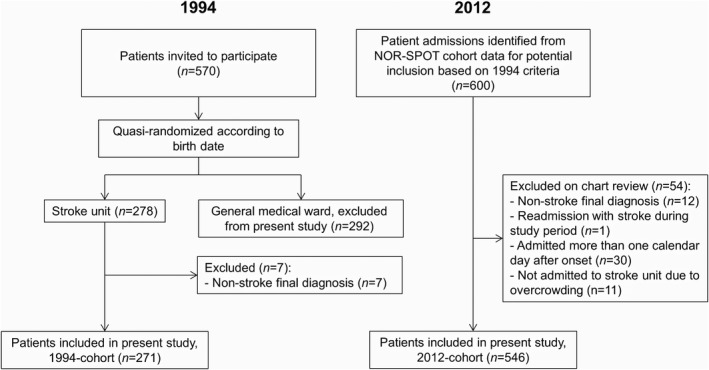
Flowchart showing inclusion and exclusion in 1994 and 2012

A total of 817 patients were included in the analysis: 271 patients in the 1994 cohort and 546 in the 2012 cohort, Figure 1. One further patient in 2012 was excluded from the survival analyses due to missing mortality data.

### 3.1 Demographics, risk factors and clinical features

**Figure 2:**
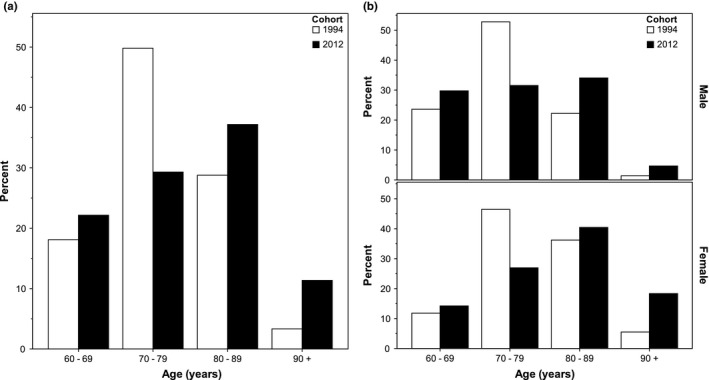
Age distribution in 1994 (*n = *271) and 2012 (*n = *546). (a) All patients, (b) males and females separately. Overall *p *< 0.001 for all between‐group comparisons, chi‐squared test

Patients in the 2012 cohort were older than in 1994 (mean age 78.8 vs. 76.7 years, *p *= 0.001), Table 1. Almost half (48.6%) of the patients in 2012 were aged 80 years and above, compared to 32.1% in 1994, Figure 2a. The shift toward higher age strata was most apparent in females, Figure 2b. Similar proportions of included patients were female, married, and living alone; however, more patients were admitted from nursing homes in 2012 (9.9% vs. 2.2%, *p *< 0.001). Smoking rates were unchanged.

**Table 1:** Patient characteristics

Characteristic	1994 (*n = *271)	2012 (*n = *546)	*p*
Age in years, mean (*SD*)	76.7 (7.4)	78.8 (9.2)	0.001
Males	75.1 (7.1)	76.3 (8.9)	0.15
Females	78.6 (7.3)	81.5 (8.8)	0.001
Female sex	127 (46.9)	267 (48.9)	0.58
Living alone	90 (33.2)	191 (35.0)	0.62
Married or partner	149 (55.0)	296 (54.2)	0.84
Admitted from nursing home	6 (2.2)	54 (9.9)	<0.001
Current smoker	60 (22.1)	106 (19.4)	0.36
Past medical history and medication use at admission			
Cerebrovascular disease	95 (35.1)	196 (35.9)	0.81
Myocardial infarction	51 (18.8)	93 (17.0)	0.53
Atrial fibrillation	58 (21.4)	184 (33.7)	<0.001
Malignancy	24 (8.9)	105 (19.2)	<0.001
Antiplatelets	71 (26.2)	256 (46.9)	<0.001
Warfarin	15 (5.5)	89 (16.3)	<0.001
Antidiabetics	31 (11.4)	77 (14.1)	0.29
Antihypertensives	152 (56.1)	380 (69.6)	<0.001
Clinical characteristics			
Intracerebral hemorrhage	38 (14.0)	79 (14.5)	0.86
Reduced consciousness	23 (8.5)	55 (10.1)	0.47
Stroke severity			
Mild	120 (44.3)	361 (66.1)	<0.001
Moderate	76 (28.0)	101 (18.5)	
Severe	75 (27.7)	84 (15.4)	
Length of stay in hospital, days			
Mean (*SD*)	9.6 (6.9)	7.5 (5.7)	<0.001
Median (IQR)	8.0 (5.0–13.0)	6.9 (4.2–9.7)	

Notes:

Values expressed as *n* (%) unless otherwise stated.

IQR: interquartile range; *SD*: standard deviation.

There were significantly more patients in 2012 with atrial fibrillation (33.7% vs. 21.4%, *p *< 0.001), malignancy (19.2% vs. 8.9%, *p *< 0.001), and using antiplatelets (46.9% vs. 26.2%, *p *< 0.001), warfarin (16.3% vs. 5.5%, *p *< 0.001), and antihypertensives (69.6% vs. 56.1%, *p *< 0.001) at admission, even after adjusting for age and sex. Patients were still more likely to be taking antiplatelets (OR: 2.90; 2.05–4.11) and warfarin (OR: 2.98; 1.62–5.48) at admission after also adjusting for relevant previous diagnosed illnesses. Rates of hypertensive prescription were no longer significantly different after these adjustments.

**Figure 3:**
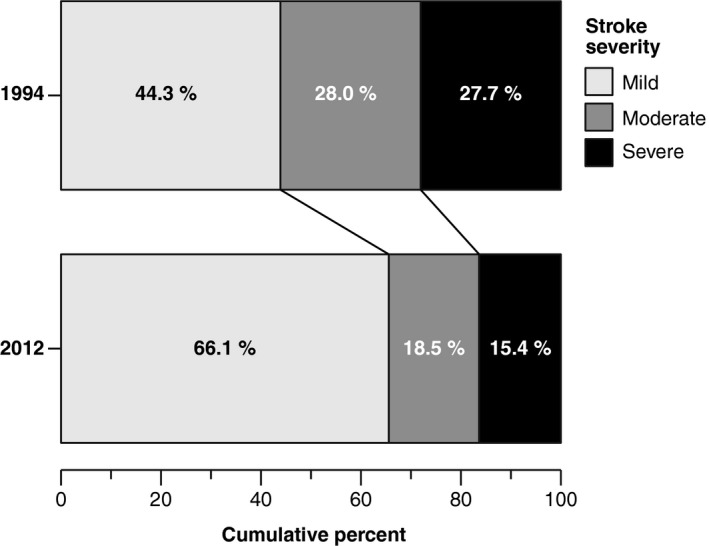
Distribution of stroke severity in 1994 (*n = *271) and 2012 (*n = *546). Overall *p *< 0.001, chi‐squared test

A similar proportion of patients were admitted with intracerebral hemorrhage and reduced consciousness; however, stroke severity was significantly milder in 2012, with a general shift toward milder strokes (Figure 3). Length of stay was shorter in 2012: median 6.9 (IQR: 4.2–9.7) versus 8.0 (IQR: 5.0–13.0), *p *< 0.001.

Data on functional independence on day 4–5 were available for 661 patients. In 2012, 46.4% were classified as independent compared to 35.7% in 1994, *p *= 0.007; however, this was no longer statistically significant after adjusting for age, sex, stroke severity and type.

### 3.2 Survival

**Figure 4:**
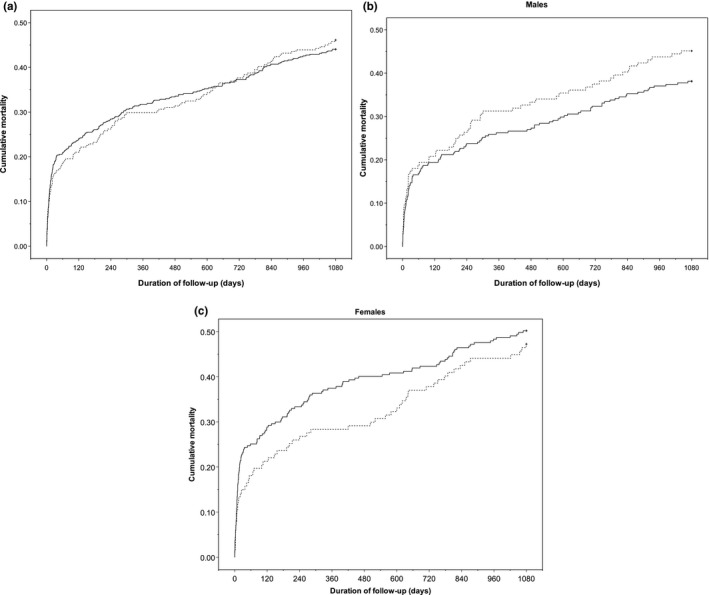
Mortality by cohort, Kaplan–Meier method. 1994 cohort (dotted line, *n* = 271), 2012 cohort (solid line, *n* = 545). (a) All patients (*p* = 0.75), (b) males (*p *= 0.17), and (c) females (*p *= 0.40). Log‐rank test

During the two distinct 3‐year observation periods, 125 patients (46.1%) in 1994 and 240 (44.0%) in 2012 died. The crude survival plots (Figure 4) show overall nonsignificant differences in survival. Adjusting for potential confounders did not change this observation; the adjusted HR for 3‐year survival in 2012 versus 1994 was 0.99 (95% CI: 0.77–1.28), see Supporting Information Table S1 for full model.

Adjusted HRs for death were estimated for different times following stroke (Table 2). Seven‐day case fatality was 8.9% in 1994 and 8.6% in 2012. At 30 days, case fatality was slightly higher in 2012 at 18.9% versus 16.2%, HR 1.68 (1.14–2.47). Similarly, the risk during days 8–30 was slightly higher in 2012, HR 1.78 (1.01–3.13). Adjusted survival was not significantly different between the cohorts beyond 30 days. Stroke severity, stroke type, and age were the most important factors independently related to mortality in the subanalysis (see Supporting Information Table S2). Early case fatality at 7 days was only predicted by stroke severity (HR: 3.59 [1.35–9.55] moderate vs. mild) and type (HR: 3.03 [1.86–4.93] ICH vs. infarct). Age became significant for survival beyond 7 days and was the only significant factor for the interval 1–3 years poststroke (HR: 1.09 [1.07–1.12]).

**Table 2:** Mortality and adjusted hazard ratios for death at times after stroke

Time point (days)	Cumulative mortality, *n* (%)		Time interval (days)	Hazard ratio, death during time interval (95% CI)
	1994 (*n = *271)	2012 (*n = *545)		
7	24 (8.9)	47 (8.6)	0–7	1.47 (0.87–2.50)
30	44 (16.2)	103 (18.9)	8–30	1.78 (1.01–3.13)
360	81 (29.9)	173 (31.7)	31–360	0.86 (0.55–1.34)
1,080	125 (46.1)	240 (44.0)	361–1,080	0.68 (0.45–1.03)

Notes:

Hazard ratios adjusted for age, sex, stroke severity, stroke type, admission from nursing home. Reference year: 1994.

CI: confidence interval.

## 4 DISCUSSION

Three‐year survival in Norwegian stroke patients aged ≥60 years treated on an SU was overall stable. Clinical characteristics and stroke‐specific risk factors changed; patients admitted in 2012 were older, more often living in supported care, and had higher comorbidity. However, they suffered milder strokes and were more often taking antiplatelets and warfarin.

Our main finding of unchanged survival appears surprising given the general decrease in stroke mortality worldwide (Feigin et al., 2014); however, secular trends in stroke case fatality within different populations are inconsistent, with some studies reporting decreasing fatality (Arboix et al., 2008; Feigin et al., 2015; Lee et al., 2011; Nimptsch & Mansky, 2014), while others report stable rates (Carrera et al., 2007; Feigin et al., 2009; Fuentes et al., 2006; Rothwell et al., 2004). The majority of studies on survival after stroke include nonhospitalized strokes (Feigin et al., 2015, 2009; Lecoffre et al., 2017; Lee et al., 2011; Numminen et al., 2000; Rothwell et al., 2004; Wieberdink et al., 2012), or strokes treated in hospital but not necessarily on an SU (Appelros et al., 2014; Arboix et al., 2008; Carrera et al., 2007; Nimptsch & Mansky, 2014). All of the patients in the present study received treatment on a comprehensive SU with consistent protocols, where the main treatment advance, thrombolysis, while improving outcomes for survivors, has not been shown to better survival (Wardlaw, Murray, Berge, & Zoppo, 2014)—and this was also observed in our population by a nonsignificant HR in the adjusted Cox regression model (Supporting Information Table S1). Thrombectomy was performed on only five patients in the 2012 cohort.

Notably, case fatality was already relatively low in 1994, considering our study populations only included patients aged ≥60 and did not exclude patients from nursing homes or with severe comorbidities. Studies in hospitalized stroke patients during the same period report in‐hospital, or 7‐day, case fatality rates in the range 7.1%–13.7%; (Appelros et al., 2014; Arboix et al., 2008; Nimptsch & Mansky, 2014) however, the mean ages of included patients were lower than in the present study, and one study (Arboix et al., 2008) included first‐ever strokes only. The low mortality in 1994 may also have contributed to our findings of unchanged survival.

Seven‐day fatality was mainly explained by stroke severity and type, and there were no between‐cohort differences. However, our data suggest a slightly higher case fatality in 2012 during days 8–30. A study using a large dataset from the Swedish stroke register Riks‐Stroke (Appelros et al., 2014) also found an increasing case fatality rate until 90 days between the years 2001 and 2010, despite a trend for less severe strokes and greater access to SU treatment. The trend appeared to be driven by patients with reduced consciousness (a proxy for stroke severity) and females. Two single‐center studies (Carrera et al., 2007; Fuentes et al., 2006) from neurological departments found nonsignificant trends for in‐hospital mortality after stroke (adjusted). They do not present survival trends beyond the acute phase.

The between‐cohort differences seen during days 8–30 could have several explanations. Withdrawal of treatment may have contributed to shorter LOS in 2012, particularly in the sickest or eldest patients. Sensitivity analyses showed that patients with severe stroke and those aged over 80 years had a greater decrease in LOS between 1994 and 2012 compared to the cohorts as a whole. This was not the case for nursing home patients (data not shown). Better and earlier diagnosis and prognostic work‐up in the later years may also have resulted in life‐prolonging treatments for the most severe strokes being withdrawn earlier in 2012 than in 1994 when a “wait and see” attitude was more common. There were significantly more nursing home patients admitted in 2012—possibly reflecting a lowered threshold for referral and/or admission. In 2012, nursing home patients could be admitted for diagnostic imaging and consideration for thrombolysis, whereas in 1994 these patients may never have been referred or admitted to hospital. This could result in an increase in prestroke morbidity that was not adequately accounted for in 2012. While we did adjust for admission from nursing home in the subanalysis, as a proxy for prestroke morbidity, we were unable to adjust for individual comorbidities in the regression models due to low events‐per‐variable. Furthermore, unmeasured confounders such as differences in complication rates, recurrent stroke, or readmissions may have differed between the cohorts.

The differences in risk profile, clinical characteristics, and demographics of patients admitted in 2012 versus 1994 are also noteworthy and may also help explain the unchanged overall survival. These may reflect real changes due to an aging stroke population, or be due to changes in diagnostic cutoffs or better screening. Stroke severity was significantly milder in 2012, with 66.1% being admitted with mild stroke, corroborating previous studies (Appelros et al., 2010; Numminen et al., 2000; Rothwell et al., 2004; Teuschl et al., 2013). This may be due to increased use of diagnostic MRI and better primary prevention; patients were more aggressively medicated in 2012 compared to 1994, also consistent with other studies (Lee et al., 2011; Rothwell et al., 2004; Wieberdink et al., 2012).

To our knowledge, our study is the first to investigate changes in survival up to 3 years among SU patients specifically. Its strengths include prospectively collected data, and virtually complete recruitment; all patients approached in 1994 consented to inclusion, and the data collection in 2012 did not require consent. The SU has had consistent treatment protocols throughout its operation. Unlike many previous studies on stroke survival, we were able to adjust for initial stroke severity.

We included specifically patients treated on the SU, and as such selection bias is always a concern. However, the study design from 1994 ensured a random allocation to the SU. In 2012, national policy was to admit all suspected strokes directly to a SU, so the sample should represent an unbiased, unselected stroke population. Few stroke patients in 2012 who met the inclusion criteria were not admitted to the SU due to overcrowding (11 admissions identified). The substantial increase to the hospital's catchment area between the study periods was due to the inclusion of three Oslo boroughs. Neither of these factors was problematic on sensitivity analyses and did not alter the regression results.

Generalizability is limited by the inclusion criteria from 1994 of patients aged ≥60, and being admitted within 24 hr. The majority of stroke patients are, however, within this age group. Although a single‐center study, the hospital's catchment population is large and diverse, represents almost 10% of Norway's population, and the hospital admits stroke patients directly and without selection. Another limitation is that we did not have data on in‐hospital complications from 1994, or events occurring after the index admission which may have influenced survival, nor were we able to analyze more than two different periods.

In conclusion, 3‐year survival among older Norwegian stroke patients treated on a comprehensive SU was overall stable despite changes to stroke management between the time periods. Survival in 1994 was already relatively high, and although the patient population in 2012 was older and had higher morbidity at admission, their stroke severity was milder and risk factors more often treated. Further investigation into these observations using data from larger cohorts of SU patients is warranted.

## DISCLOSURES

None.
