# Supplementary material for: Changes in survival and characteristics among older stroke unit patients—1994 versus 2012
Source: Brain Behav. 2018 Nov 25;9(1):e01175. doi: 10.1002/brb3.1175 (PMC6346673; doi:10.1002/brb3.1175)
Supplement: Supplementary file 1 [file BRB3-9-e01175-s001.docx]

**Table S1** Full Cox regression model for three-year survival showing variables included in the final model. CI = confidence interval

| **Variable** | **Hazard ratio (95% CI)** |
| --- | --- |
| Cohort (reference: 1994) | 0.99 (0.77 – 1.28) |
| Age | 1.08 (1.06 – 1.09) |
| Female sex | 0.85 (0.67 – 1.06) |
| Stroke severity  Mild  Moderate  Severe | Reference  2.11 (1.60 – 2.78)  4.82 (3.64 – 6.37) |
| Intracerebral hemorrhage | 1.96 (1.49 – 2.58) |
| Current smoker | 1.27 (0.94 – 1.70) |
| Cerebrovascular disease | 1.18 (0.94 – 1.47) |
| Myocardial infarction | 1.30 (1.00 – 1.70) |
| Atrial fibrillation | 1.27 (1.01 – 1.60) |
| Malignancy | 1.17 (0.89 – 1.54) |
| Antidiabetics | 1.74 (1.28 – 2.38) |
| Antihypertensives | 1.05 (0.83 – 1.34) |
| Admitted from nursing home | 2.13 (1.52 – 2.99) |
| Thrombolysis | 0.91 (0.55 – 1.51) |
