# Supplementary material for: Changes in survival and characteristics among older stroke unit patients—1994 versus 2012
Source: Brain Behav. 2018 Nov 25;9(1):e01175. doi: 10.1002/brb3.1175 (PMC6346673; doi:10.1002/brb3.1175)
Supplement: Supplementary file 2 [file BRB3-9-e01175-s002.docx]

**Table S2** Cox regression models for hazard ratios for death during different time intervals after stroke showing variables included. Reference: 1994 cohort, mild stroke severity. CI = confidence interval

| **Time interval**  **(days)** | **Variable** | **Hazard ratio**  **(95% CI)** |
| --- | --- | --- |
| 0 – 7 | Cohort | 1.47 (0.87 – 2.50) |
|  | Age | 1.01 (0.98 – 1.04) |
|  | Female sex | 0.76 (0.47 – 1.24) |
|  | Stroke severity  Moderate  Severe | 3.59 (1.35 – 9.55)  24.06 (10.50 – 55.14) |
|  | Intracerebral hemorrhage | 3.03 (1.86 – 4.93) |
|  | Admission from nursing home | 1.07 (0.56 – 2.07) |
| 8 – 30 | Cohort | 1.78 (1.01 – 3.13) |
|  | Age | 1.09 (1.06 – 1.12) |
|  | Female sex | 0.78 (0.48 – 1.27) |
|  | Stroke severity  Moderate  Severe | 8.60 (3.98 – 18.56)  18.49 (8.52 – 40.16) |
|  | Intracerebral hemorrhage | 1.86 (1.00 – 3.46) |
|  | Admission from nursing home | 1.68 (0.94 – 3.02) |
| 31 – 360 | Cohort | 0.86 (0.55 – 1.34) |
|  | Age | 1.09 (1.06 – 1.12) |
|  | Female sex | 0.76 (0.51 – 1.13) |
|  | Stroke severity  Moderate  Severe | 1.38 (0.85 – 2.24)  2.71 (1.60 – 4.58) |
|  | Intracerebral hemorrhage | 1.16 (0.63 – 2.14) |
|  | Admission from nursing home | 3.28 (1.82 – 5.90) |
| 361 – 1080 | Cohort | 0.68 (0.45 – 1.03) |
|  | Age | 1.09 (1.07 – 1.12) |
|  | Female sex | 0.87 (0.59 – 1.29) |
|  | Stroke severity  Moderate  Severe | 1.55 (0.99 – 2.44)  0.99 (0.48 – 2.03) |
|  | Intracerebral hemorrhage | 1.30 (0.74 – 2.29) |
|  | Admission from nursing home | 2.24 (0.93 – 5.37) |
